# Supplementary material for: Content-rich biological network constructed by mining PubMed abstracts
Source: BMC Bioinformatics. 2004 Oct 8;5:147. doi: 10.1186/1471-2105-5-147 (PMC528731; doi:10.1186/1471-2105-5-147)
Supplement: Additional File 2 — The original results of the above study (non-essential files are deleted to keep the file size under the limit set by BMC bioinformatics). [file 1471-2105-5-147-S2.bz2 › chilibotAdditionalFile2/dip10/174ID9461436E589/html/E4TF1A_GABPB1.html]

 


 **E4TF1A** and **GABPB1** 
  
Found 8 abstracts in PubMed, retrieved 8.  
 

 What does Google say? 
 PDF only 
| .edu only 

---

**Interactive relationship** (e.g. stimulation, inhibition, etc)

**Neutral relationship**- The 48 amino acids at the E4TF1  [ **GABPB1** ]  60  [ **E4TF1A** ]  C terminus were required for interaction with the other type of subunit.  Ref: 8137823 EMBO J, 1994
- Transcription factor E4TF1  [ **GABPB1** ]  is composed of two types of subunit, an ets related DNA binding protein, E4TF1  [ **GABPB1** ]  60  [ **E4TF1A** ] , and its associated proteins with four tandemly repeated Notch ankyrin motifs, E4TF1 53  [ **GABPB1** ]  and E4TF1  [ **GABPB1** ]  47.  Ref: 8137823 EMBO J, 1994

**Non-interactive relationship** (e.g. studied together, co-existance, homology, etc.)

- When RBF 1 site was used for sequence specific DNA affinity purification from erythroleukemia cells, reconstitution assays, immunoblotting analysis and peptide mapping show that the two major co purified proteins are identical to human E4TF1  [ **GABPB1** ]  60  [ **E4TF1A** ]  and 53 proteins.  Ref: 8208529 Oncogene, 1994
- We analyzed the localization of the E4TF1  [ **GABPB1** ]  60  [ **E4TF1A** ]  gene in more detail by genomic Southern hybridization and determined the sequence of the exons and the regions surrounding the intron boundaries.  Ref: 8543189 Gene, 1995
- Assignment of the E4TF1  [ **GABPB1** ]  60  [ **E4TF1A** ]  gene to human chromosome 21q21.2 q21.3.  Ref: 8543189 Gene, 1995
- We report here that E4TF1  [ **GABPB1** ]  60  [ **E4TF1A** ]  locates in the long arm of chromosome 21 at q21.2 q21.3 and contains a total of ten exons.  Ref: 8543189 Gene, 1995
- The predicted amino acid sequence of each cDNA clone revealed that E4TF1  [ **GABPB1** ]  60  [ **E4TF1A** ]  had an ETS domain, which is a DNA binding domain common to ets related transcription factors.  Ref: 8441384 Mol Cell Biol, 1993
- Anti E4TF1  [ **GABPB1** ]  60  [ **E4TF1A** ]  antibody generated a supershifted complex.  Ref: 11790771 J Biol Chem, 2002
- Heterodimers of E4TF1  [ **GABPB1** ]  47 and E4TF1  [ **GABPB1** ]  60  [ **E4TF1A** ]  weakly stimulated transcription in vitro.  Ref: 8137823 EMBO J, 1994
- One trapped sequence showed complete homology with nucleotide sequence D13318 of GenBank, which corresponds to the gene for human transcription factor E4TF1  [ **GABPB1** ]  60  [ **E4TF1A** ]  HGMW approved nomenclature GABPA.  Ref: 7590737 Genomics, 1995
- Furthermore, coexpression of E4TF1  [ **GABPB1** ]  60  [ **E4TF1A** ]  and E4TF1 53  [ **GABPB1** ]  markedly increased aurora A promoter activity.  Ref: 11790771 J Biol Chem, 2002
- In this study, we identified a new E4TF1  [ **GABPB1** ]  subunit, designated E4TF1  [ **GABPB1** ]  47, which has no DNA binding activity but can associate with E4TF1  [ **GABPB1** ]  60  [ **E4TF1A** ] .  Ref: 8441384 Mol Cell Biol, 1993
- Mapping of the human transcription factor GABPA E4TF1  [ **GABPB1** ]  60  [ **E4TF1A** ]  gene to chromosome 21.  Ref: 7590737 Genomics, 1995
- They interacted with E4TF1  [ **GABPB1** ]  60  [ **E4TF1A** ]  through the N terminal flanking region to form a heterodimer.  Ref: 8137823 EMBO J, 1994
- Therefore, heterodimers composed of E4TF1 53  [ **GABPB1** ]  and E4TF1  [ **GABPB1** ]  60  [ **E4TF1A** ]  were further dimerized, resulting in the formation of a tetrameric complex, which stimulated transcription in vitro.  Ref: 8137823 EMBO J, 1994
- The gene encoding human transcription factor E4TF1  [ **GABPB1** ]  60  [ **E4TF1A** ]  was previously mapped to chromosome 21q21.  Ref: 8543189 Gene, 1995
- Furthermore, co expression of E4TF1  [ **GABPB1** ]  60  [ **E4TF1A** ]  and E4TF1 53  [ **GABPB1** ]  47 squelched TPO gene expression in PLC and HepG2 cells.  Ref: 9111044 J Biol Chem, 1997
- Anti E4TF1  [ **GABPB1** ]  60  [ **E4TF1A** ]  GABPalpha or anti E4TF1 53  [ **GABPB1** ]  47 GABPbeta antibodies supershifted the complex in gel shift assay.  Ref: 9111044 J Biol Chem, 1997
- In addition to APP, the cosmid contains the E4TF1  [ **GABPB1** ]  60  [ **E4TF1A** ]  transcription factor, the U2AF35 pre mRNA splicing factor, and the cystathionine beta synthetase CBS gene.  Ref: 9931491 Gene, 1999
- The introns of APP and E4TF1  [ **GABPB1** ]  60  [ **E4TF1A** ]  are 49 and 24 fold smaller in Fugu than in human, and the intergenic distance is compressed at least 100 fold.  Ref: 9931491 Gene, 1999
- E4TF1  [ **GABPB1** ]  60  [ **E4TF1A** ]  bound to DNA as a monomer.  Ref: 8137823 EMBO J, 1994
- APP and E4TF1  [ **GABPB1** ]  60  [ **E4TF1A** ]  map within 21q21.  Ref: 9931491 Gene, 1999
